# Environmental colonization and onward clonal transmission of carbapenem-resistant *Acinetobacter baumannii* (CRAB) in a medical intensive care unit: the case for environmental hygiene

**DOI:** 10.1186/s13756-018-0343-z

**Published:** 2018-04-10

**Authors:** Deborah H. L. Ng, Kalisvar Marimuthu, Jia Jun Lee, Wei Xin Khong, Oon Tek Ng, Wei Zhang, Bee Fong Poh, Pooja Rao, Maya Devi Rajinder Raj, Brenda Ang, Partha Pratim De

**Affiliations:** 1 Institute of Infectious Diseases and Epidemiology, Tan Tock Seng Hospital, 11 Jalan Tan Tock Seng, Singapore, 304833 Singapore; 2 National University of Singapore, 21 Lower Kent Ridge Road, Singapore, 119077 Singapore; 3 Infection Control Unit, Tan Tock Seng Hospital, 11 Jalan Tan Tock Seng, Singapore, 304833 Singapore; 4 Department of Laboratory Medicine, Tan Tock Seng Hospital, 11 Jalan Tan Tock Seng, Singapore, 304833 Singapore

**Keywords:** Colonization, Carbapenem-resistant *Acinetobacter baumannii*, Intensive care unit, Whole genome sequencing

## Abstract

**Background:**

In May 2015, we noticed an increase in carbapenem-resistant *Acinetobacter baumannii* (CRAB) infections in the Medical Intensive Care Unit (MICU). To investigate this, we studied the extent of environmental contamination and subsequent onward clonal transmission of CRAB.

**Methods:**

We conducted a one-day point prevalence screening (PPS) of the patients and environment in the MICU. We screened patients using endotracheal tube aspirates and swabs from nares, axillae, groin, rectum, wounds, and exit sites of drains. We collected environmental samples from patients’ rooms and environment outside the patients’ rooms. CRAB isolates from the PPS and clinical samples over the subsequent one month were studied for genetic relatedness by whole genome sequencing (WGS).

**Results:**

We collected 34 samples from seven patients and 244 samples from the environment. On the day of PPS, we identified 8 CRAB carriers: 3 who screened positive and 5 previously known clinical infections. We detected environmental contamination in nearly two-thirds of the rooms housing patients with CRAB. WGS demonstrated genetic clustering of isolates within rooms but not across rooms. We analysed 4 CRAB isolates from clinical samples following the PPS. One genetically-related CRAB was identified in the respiratory sample of a patient with nosocomial pneumonia, who was admitted to the MICU five days after the PPS.

**Conclusion:**

The extensive environmental colonization of CRAB by patients highlights the importance of environmental hygiene. The transmission dynamics of CRAB needs further investigation.

## Background

Carbapenem-resistant *Acinetobacter baumannii* (CRAB) has been emerging as a healthcare threat in recent years, owing to its ability to survive in the environment for prolonged periods and to acquire multiple antibiotic resistance mechanisms [1, 2]. There is a high prevalence of *A. baumannii* in the Asia-Pacific region, with hospitals reporting rates of CRAB infection from 50% to over 90% in countries such as Vietnam and Singapore [3]. A survey of multidrug-resistant organisms (MDROs) across public hospitals in Singapore in 2006 found that the incidence density of all CRAB isolates was 0.59 per 1000 inpatient-days, while the incidence density in the intensive care unit (ICU) increased more than 4-fold to 2.78 per 1000 ICU inpatient-days [4]. More recently, Cai et al. reported that 71.9% of all *Acinetobacter* spp. implicated in healthcare-associated infections were carbapenem non-susceptible [5].

Point-prevalence studies have been useful in investigating the extent of colonization with MDROs in various healthcare settings [6]. An investigation into an outbreak of *A. baumannii* in an ICU found that environmental colonization was present in 39.3% of swabs taken, of which 7.1% had similar antibiotic sensitivity profiles to the outbreak strain [7]. Another study investigating an outbreak of *Pseudomonas aeruginosa* in a neonatal ICU demonstrated that although majority of the clinical isolates were identical, the additional use of whole genome sequencing (WGS) demonstrated that some of the isolates were not in fact related to the outbreak strain despite having the same susceptibility pattern [8]. These studies highlight the limitation of using the phenotypic method in defining relatedness of isolates.

In May 2015, the prevalence of CRAB infections in the medical intensive care unit (MICU) of a teaching hospital in Singapore increased from a baseline rate of about 0.32 cases per 1000 patient days, to 0.55 cases per 1000 patient days. To investigate the surge, we conducted this study to (i) estimate the prevalence of CRAB among patients in the MICU, (ii) identify the level of environmental contamination within the unit and (iii) investigate the role of environmental contamination in horizontal transmission of CRAB.

## Methods

### Setting

We conducted a one-day point prevalence screening (PPS) and one-month surveillance of clinical cultures for CRAB in the MICU of Tan Tock Seng Hospital, a teaching hospital in Singapore. The hospital has 1500 beds and houses four intensive care units, namely the MICU, Surgical Intensive Care Unit, Neurological Intensive Care Unit and the Coronary Care Unit. The MICU contains a total of 6 high-dependency care beds and 12 single-room intensive care beds, of which 3 are negative- pressure rooms.

### Study procedures

**Fig. 1:**
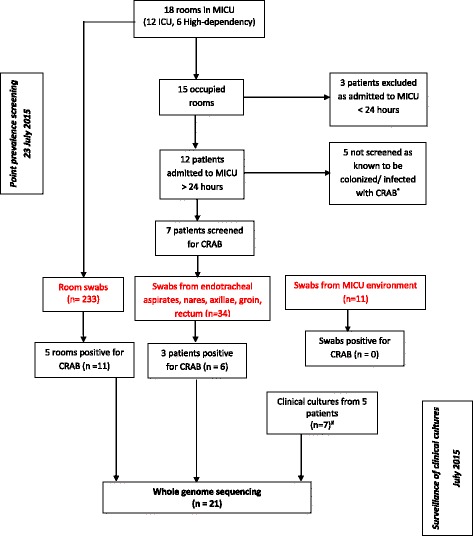
Flowchart of swabs collected from the MICU. Legend: *MICU*, medical intensive care unit; *CRAB* carbapenem-resistant *Acinetobacter baumannii*, *PPS* point prevalence screening, *n* = number of swabs. *All isolates were not available for sequencing. ^#^4 out of the 7 isolates were available for sequencing

The study comprised two components: the first was a one-day PPS of patients who were in the MICU on the day of screening (23 July 2015); while the second component was a one-month surveillance of clinical cultures of CRAB isolated from patients within the MICU in the month of July 2015 (Fig. 1).

During the PPS, we collected patients’ endotracheal tube (ETT) aspirates and swabs from their nares, axillae, groin, wounds and exit sites of drains, if any. Swabs were not taken from patients who had been in the MICU for less than 24 h or who were known CRAB carriers.

Environmental swabs were taken from all the patient rooms in the MICU, regardless of whether they were occupied by patients at the time. The following surfaces were swabbed: physiological monitor terminals, bedside rails, automatic door buttons, infusion pumps, resuscitation bags, ventilator panels, ventilator humidifiers, suction bottles, stethoscopes, patients’ lockers, all-purpose tables, sink faucet openings, and sink drainage sites. Swabs were also taken from the MICU environment outside the patients’ rooms. This included the computer keyboards and ‘mice’, cleaned ventilators, equipment carts and emergency carts.

We followed the PPS with a one-month surveillance for clinical cultures to detect onward clonal transmission. The Department of Laboratory Medicine cultured, identified and stored all CRAB isolates from existing and newly admitted MICU patients.

### Clinical data collection

We investigated genetic linkage by collecting data from electronic medical records on ward movements of involved patients, overlap of healthcare workers caring for the patients during their stay in the MICU and sharing of any medical equipment.

### Specimen collection and culture

All samples were collected by two microbiology doctors and eight infection control nurses who were trained and supervised by the microbiology doctors. Sterile cotton swabs were moistened with sterile water and were rolled over the surface five times. Each swab was collected in Amie’s transport media. The samples were inoculated onto MacConkey agar and incubated at 37 °C for 48 h. A meropenem disc (10 μg, BD Diagnostics, USA) was placed on each plate to select for carbapenem-resistant organisms. Matrix-assisted laser desorption/ionization time-of-flight mass spectrometry (MALDI-TOF MS, Bruker Daltonics, GmbH, Germany) was performed on colonies growing next to the meropenem disc after overnight incubation to identify CRAB*.* Susceptibility testing was performed on all isolates of *A. baumannii* using the Kirby –Bauer method and those with a meropenem zone ≤14 mm were interpreted as resistant based on the Clinical Laboratory and Standards Institute criteria (M100- S22) [9].

### Whole genome sequencing, species identification and strain typing

Sequencing libraries for each isolate were prepared according to the manufacturer’s recommendation using the Illumina Nextera XT kit (Illumina Inc., USA). The Illumina FastQ files are in the process of being deposited into the GenBank database. De novo assembly of the Illumina reads was performed using SPAdes Genome Assembler [10]. Bacterial species were identified using Kraken [11]. Multilocus STs were identified using SRST2 [12]. Isolates with species discordance comparing phenotypic with genotypic speciation were excluded from further analysis.

### Bacterial core genome analysis and determination of transmission clusters

The program Parsnp was used to generate core genome alignments (i.e. conserved orthologous regions present in all included genomes) for all isolates [13]. As previously described, Parsnp screens and excludes sequences based on a threshold MUMi distance. Input for this alignment was the de novo assemblies from the bacterial isolates, as well as a reference sequence available from GenBank (Accession Number: NC_009085.1). The core nucleotide alignments were used as input for Gubbins to exclude putative single-nucleotide polymorphisms (SNPs) arising from recombination [14]. Sequences were then aligned to be concatenated into an artificial ‘genome assembly’, which was used to create a Maximum Likelihood phylogenetic tree with 100 replicates for bootstrap value calculation.

To determine transmission clusters of highest confidence, we defined transmission clusters as isolates that had pair-wise SNP distance less than the pair-wise SNP threshold [15]. The pair-wise SNP threshold was defined as the maximum intra-patient SNP count comparing all repeated isolates with identical species and strain type from the same patient [16]. The pair-wise SNP threshold implemented for this study based on the maximum pair-wise SNP count between CRAB isolates isolated from the same patient (excluding environmental samples) was equal or less than 11 SNP.

## Results

**Fig. 2:**
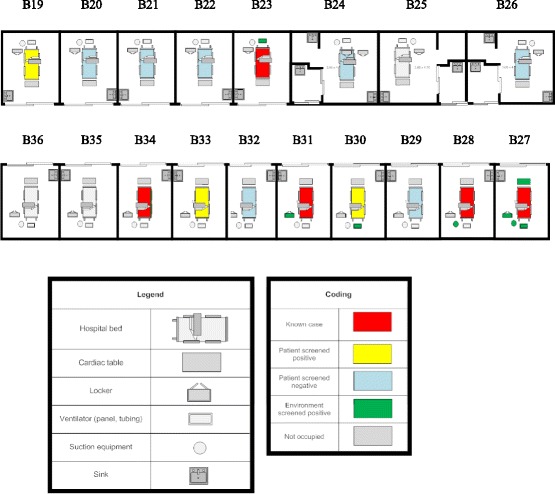
Floor plan of the MICU showing the distribution patients and CRAB

There were fifteen patients in the MICU on the day of screening, with three unoccupied rooms. (Figure 2) Five patients were not swabbed as they were known to have CRAB, while another three were excluded from screening as they had been admitted to the MICU for less than 24 h. We collected 34 swabs from the remaining seven patients, 233 swabs from the environment of all 18 rooms in the MICU and 11 swabs from the external environment as demonstrated in Fig. 1.

Three of the 7 screened patients were found to be colonized with CRAB, identified from six surveillance swabs (1 ETT aspirate, 2 nasal swabs, 2 axillary swabs and 1 groin swab). All the patients who were colonised, infected or screened positive for CRAB were mechanically ventilated. Of the eight rooms occupied by patients colonized or infected with CRAB, there was environmental contamination present in 28% (5/18) of the rooms. None of the swabs taken from door switches, sinks or faucet openings were positive for CRAB. The 17 positive screening swabs were sent for sequencing. All the swabs from the external environment were negative for CRAB.

Within the one-month period of PPS, CRAB was isolated from 7 clinical cultures from 5 patients. Of these, 4 isolates from 4 patients were available and sent for sequencing.

### WGS results and clinical correlation

**Fig. 3:**
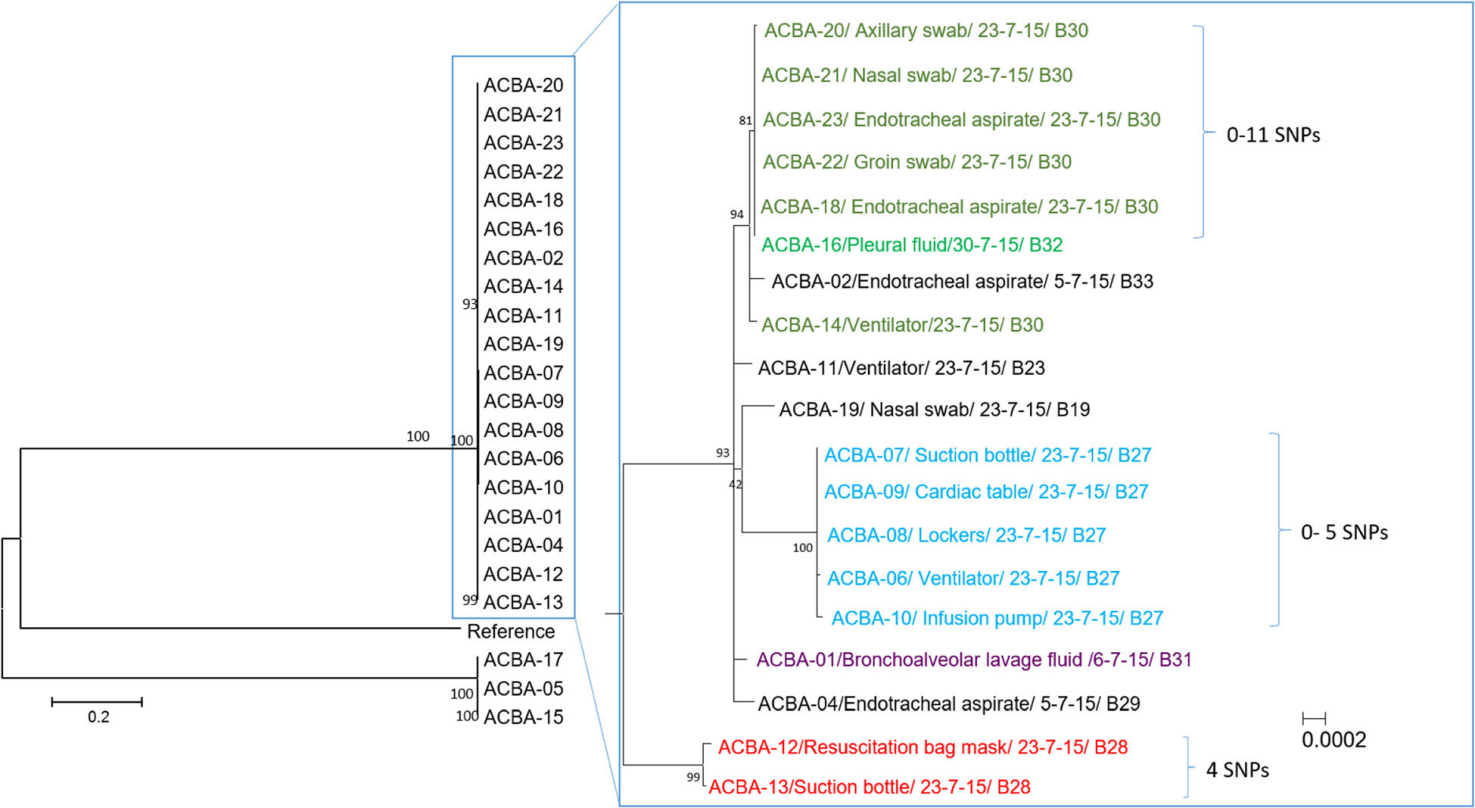
Whole genome sequencing of clinical and screening isolates of CRAB. Labels were configured to represent: Specimen number/specimen type/date of collection/bed number. Legend: *ACBA Acinetobacter baumannii, SNPs* single nucleotide polymorphisms

A total of 21 isolates were sent for WGS. Results of the WGS (Fig. 3) demonstrated that among patients whose rooms were colonised with CRAB, the isolates from the patient were clonal with their environment but differed between rooms. (Figure 2) Although isolates ACBA-6, 7 and 10 from bed (B) 27 showed intermediate susceptibility to ampicillin-sulbactam, while isolates ACBA-8 and 9 were pan-resistant, there was no other genotypic-phenotypic speciation discordance as shown by the antimicrobial susceptibility testing results in Table 1.

**Table 1:** Antibiotic susceptibility of isolates sent for WGS

	Swab	Ceftazidime	Cefepime	Ampicillin-sulbactam	Piperacillin-tazobactam	Meropenem	Gentamicin	Amikacin	Ciprofloxacin	Cotrimoxazole
ACBA 1	Locker	R	R	R	R	R	R	R	R	R
ACBA 2	Endotracheal tube aspirate	R	R	I	R	R	R	R	R	R
ACBA 4	Pleural fluid	R	R	R	R	R	R	R	R	R
ACBA 6	Ventilator panel	R	R	I	R	R	R	R	R	R
ACBA 7	Suction bottle connector	R	R	I	R	R	R	R	R	R
ACBA 8	Locker	R	R	R	R	R	R	R	R	R
ACBA 9	All-purpose table	R	R	R	R	R	R	R	R	R
ACBA 10	Infusion pump	R	R	I	R	R	R	R	R	R
ACBA 11	Heat and moisture exchanger	R	R	R	R	R	R	R	R	R
ACBA 12	Resuscitation bag-face mask connector valve	R	R	R	R	R	R	R	R	S
ACBA 13	Suction bottle connector	R	R	R	R	R	R	R	R	S
ACBA 16	Pleural fluid	R	R	R	R	R	R	R	R	R
ACBA 17	Axilla	R	R	R	R	R	R	R	R	R
ACBA 14	Ventilator panel	R	R	R	R	R	R	R	R	R
ACBA 18	Tracheal aspirate	R	R	R	R	R	R	R	R	R
ACBA 19	Nasal	R	R	R	R	R	R	R	R	R
ACBA 20	Axilla	R	R	R	R	R	R	R	R	R
ACBA 21	Nasal	R	R	R	R	R	R	R	R	R
ACBA 22	Groin	R	R	R	R	R	R	R	R	R
ACBA 23	Ventilator humidifier	R	R	R	R	R	R	R	R	R

The pair-wise SNP threshold was as follows (Fig. 3):

- B27: ACBA-6, ACBA-7, ACBA-8, ACBA-9, ACBA-10; 0–5 SNP.
- B28: ACBA-12, ACBA-13; 4 SNP.
- B30: ACBA-18, ACBA-20, ACBA-21, ACBA-22, ACBA-23; 0–11 SNP.

One of the 4 CRAB clinical isolates from B32 (ACBA-16) collected during the one-month surveillance revealed genetic relatedness with CRAB isolates from the patient and environment in B30, with a pair-wise SNP of one nucleotide. They had similar carbapenem resistance mechanisms (blaOXA-23). Further investigations showed that patient from B30 had been in the MICU for 54 days and was screened positive during the PPS. The other patient was admitted to the MICU after the screening and had been in the MICU for 3 days before developing pneumonia due to CRAB. These two patients were both in the MICU between 28 and 30 July 2015 but did not have previous admissions in the hospital that overlapped in time. Medical equipment were not shared between them; however, there was overlap of healthcare workers (two doctors and one nurse) who cared for both patients during this 3-day period.

## Discussion

This one-day PPS found that the overall prevalence of CRAB in the MICU was 53% (8/15 patients). Environmental colonization was present in over a quarter of the room in the MICU, but only present in rooms occupied by patients with CRAB. We also found evidence for onward clonal transmission of CRAB; however, the exact mode of transmission needs further investigation.

There appears to be a correlation to the proximity of the equipment to the patient as items such as ventilators, control panels, infusion pumps and bedrails were more likely to be contaminated. This may be due to the fact that intubated patients may be undergoing aerosol-generating procedures or suctioning. A recent study also found that nebulized medication administration (NMA) and bronchoscope with NMA generated significantly higher levels of aerosols, compared to other activities such as bronchoscopy alone or non-invasive ventilation [17]. In contrast, door switches, sinks or faucet openings, which were situated further away from the head of the bed, were all negative for CRAB, as were the swabs from the external environment. In addition, the areas positive for CRAB were also high-touch surface areas, as defined by Carling et al [18]. All environmental surfaces are currently cleaned with granular sodium dichloroisocyanurate (NaDCC) diluted to 5000 ppm (ppm) as well as Mikro Quat® once a week. High-touch surface areas are cleaned on a daily basis while low-touch surface areas are cleaned when the patients are transferred out of the MICU. Reusable equipment such as infusion pumps and oximetry probes are wiped down with alcohol wipes after each patient use. As each patient has their own dedicated blood pressure cuff, they are cleaned with alcohol wipes after the patient is transferred out of the MICU.

According to local infection control policy, any staff member entering a patient’s room is required to don personal protective equipment prior to entering and to doff prior to exiting the room, regardless of whether the patient is known to have colonization or infection with any MDR organisms. In the MICU, alcohol-based hand rub is placed at the foot of the patient’s bed in close proximity to the exit, which facilitates the use of the hand rub prior to exiting the room. This may explain why the door switches and external environment were negative for CRAB. Staff are also encouraged to do hand hygiene with alcohol-based hand rub rather than water with disinfectant soap. Following the one-day PPS, strict contact precautions were enforced for patients known to be colonized or infected with CRAB. Surveillance rates of CRAB over the next 3 months reduced to the baseline average of 0.38 cases per 1000-patient days.

Although *A. baumannii* is ubiquitous in the environment, it is known to have a predilection for water sources [19]. A study conducted in an ICU in Japan reported that an outbreak of *A. baumannii* had been caused by tap water from sinks colonized with the organism and was transmitted through oral care using tap water [19]. In our study, all the swabs taken from sink faucet openings and drain holes were negative for CRAB. This may be due to local infection control procedures which prohibit staff from discarding body fluids or water that has been used for the patient down the sink. In the MICU, all contaminated fluid is disposed of in the sluice. However, compliance to local policies also need to be confirmed with audits of disposal methods and adherence to hand hygiene.

Similarly, our study also found that although antibiotic susceptibility of our isolates were all similar, WGS found that majority of them were not related across rooms. This shows that it is insufficient to rely on phenotypic patterns alone in determining relatedness of the isolates. WGS was instrumental in identifying one case of linked transmission between two patients in the MICU. The two patients did not share a room or medical equipment in the MICU. However, three healthcare workers had close contact with both patients. This occurred despite the background hand hygiene compliance rate averaging 72.5% and the mean compliance to contact precautions at 77.5% for the year of 2015. A CRAB outbreak investigation in a Korean ICU 10.9% of healthcare workers’ hands were colonised with CRAB, suggesting that healthcare workers may inadvertently be responsible for horizontal transmission within the ICU [20]. Longitudinal studies, similar to those conducted by Price et al. [21] for MRSA, may help define the modes of transmission of CRAB and facilitate design of appropriate infection prevention and control measures.

This study demonstrates the limitations of a one-day PPS in investigating horizontal transmission, since the PPS only provides a snapshot of the extent of the spread of CRAB within the MICU. If the additional clinical isolate had not been sent for WGS, it would have likely been concluded that there was no horizontal transmission within the MICU. Further surveillance is required to understand the transmission dynamics and extent of horizontal transmission, as well as compliance rates to the use of personal protective equipment.

Another limitation of the study was the exclusion of patients who had been admitted for less than 24 h to the MICU which may have resulted in selection bias and underestimated the CRAB prevalence in the MICU by omitting patients who were admitted with CRAB. However, this is less likely to underestimate the rate of CRAB acquisition in the MICU as CRAB acquisition during such a short period of time would be rare.

One strength of the study is the large number of screening samples collected from each patient and the environment, with an average of five swabs collected from each patient. In a study of patients who were known to be carriers of multidrug-resistant *A.baumannii* and underwent screening for carriage from six sites (nostrils, pharynx, skin, rectum, wounds and endotracheal aspirates), the sensitivity of a single site and the sensitivity of six sites was only 55% [22]. Another study which surveyed 160 ICU patients who had at least one positive clinical culture found that a combination of four swabs from tracheal aspirates, rectum, sternal skin and urine identified up to 85% of patients known to have CRAB [23]. We also took nasal swabs from our patients, which were positive in two of the three patients screened to be positive for CRAB, suggesting that this may also be an important screening site. As an additional 20% of patients were found to be carriers of CRAB from this one-day surveillance screening alone, our findings also suggest that there may be a need to carry out regular screening of patients on admission and discharge from the MICU to identify any ongoing transmission.

## Conclusion

In conclusion, this study demonstrates that in a setting where CRAB is endemic, environmental contamination is common if patients are also colonized or infected with CRAB. Furthermore, longitudinal screening of patients in an environment where patients are at high risk for acquisition of CRAB, combined with WGS can be a powerful tool in discerning nosocomial CRAB transmission and informing infection control decisions. We are currently piloting the use of real-time WGS combined with epidemiological linkage analysis to track the transmission of CRAB as they emerge, to allow for early institution of infection control measures and reducing further transmission.

## Abbreviations

B: Bed
CRAB: Carbapenem-resistant *Acinetobacter baumannii*
ETT: Endotracheal tube
ICU: Intensive Care Unit
MDRO: Multi-drug resistant organisms
MICU: Medical Intensive Care Unit
NaDCC: sodium dichloroisocyanurate
NMA: Nebulised medication administration
ppm: parts per million
PPS: Point prevalence screening
SNP: Single-nucleotide polymorphism
WGS: Whole genome sequencing
